# Palliative care patients in the emergency medical service: a retrospective cohort study from Finland

**DOI:** 10.1186/s12913-024-10905-4

**Published:** 2024-04-29

**Authors:** Eemil Pesonen, Pauli Vuorinen, Leena Surakka, Juho T. Lehto, Sanna Hoppu

**Affiliations:** 1 Faculty of Medicine and Health Technology, Tampere University, Tampere, Finland; 2 Emergency Medical Services, Centre for Prehospital Emergency Care, Department of Emergency, Anaesthesia and Pain Medicine, Tampere University Hospital, Tampere, Finland; 3 Institute of Public Health and Clinical Nutrition, University of Eastern Finland, Kuopio, Finland; 4 Siun Sote - North Karelia Social and Health Services Joint Authority, Palliative Care Centre, Joensuu, Finland; 5 Palliative Care Centre, Tampere University Hospital, Tampere, Finland

**Keywords:** Emergency medical services, Palliative, End-of-life

## Abstract

**Background:**

Paramedics are often involved in treating palliative care patients with difficulties regarding symptom control. They report minimal training in palliative care and find decision-making difficult. This often leads to overtreatment and unnecessary transportation to the emergency department. The study’s objective is to determine how much palliative patients use emergency services, how well are they recognized by paramedics and how paramedics choose care in terms of treatment and transportation.

**Methods:**

This study is a retrospective cohort study based in the Finnish Tampere University Hospital area. We included patients with a palliative care decision setting the goal of therapy as palliative intent between 1 August 2021 and 31 December 2021 and who died before 1 April 2022. From these patients, records of nurse paramedic visits were retrieved. Descriptive statistics were used to describe the data.

**Results:**

Paramedics visited 69 patients in 97 callouts. These callouts comprised 0.26% of the total dispatches in the study area. The most common reasons for callouts were general weakness, breathing difficulty and pain. The paramedics provided treatment in 40% of the missions. 55% of the patients were transported to the emergency department. A palliative care plan was recognized by the paramedics in 42 of the 97 callouts. A total of 38 patients were recognized as palliative care patients by the paramedics while in the cases of 31 patients, palliative care was not recognized in any dispatch.

**Conclusion:**

Patients in palliative care cause only a minimal load on the emergency medical services, but the paramedics do not necessarily recognize them as such. This leads to the risk of overtreatment and a high transportation rate to the emergency department, which is not an ethical choice. Recognition and treatment provided to palliative care patients by the paramedics could be improved with additional training and greater availability of patient records.

## Background

The population in Europe is aging. It is estimated that consequently the demand for palliative care services will increase rapidly in the following decades [1]. The World Health Organization states that patients who are in the end-of-life stage should be able to choose their preferred place to spend their final days and eventually to succumb [2]. Most prefer to be treated and to die at home [3]. Nevertheless, patients are often transferred to a hospital when their condition declines [3, 4].

Anticipation is the key element in palliative care although decline of the condition and lack of symptom control may still emerge unexpectedly [5]. Paramedics are often involved in these situations as immediate response after an emergency call, especially out of hours [6]. Paramedics are typically more familiar with life-sustaining treatments than with symptom control and report minimal training on palliative care [4]. It is recognized that they experience difficulties making decisions about the treatment and possible transportation of a palliative patient. Lack of adequate information and time pressure often lead to a situation in which paramedics see transportation to the emergency department as their only option [7, 8]. This, however, is not in line with palliative care principles. Unnecessary transitions may deteriorate quality of life and expose palliative care patients to futile medical interventions and tests [9, 10].

Recent research details the most common causes of paramedic visits to palliative care patients [4]. It remains unclear whether the paramedics recognize the palliative patients in the field. Difficulties in obtaining information about the palliative care plan could explain the high numbers of transported patients.

Our aim is to recognize the extent to which the patients in palliative care use emergency medical services, whether nurse paramedics identify palliative care patients during the callout, and how they choose care in terms of treatment and transportation according to palliative care principles.

## Methods

### Study design

This is a retrospective cohort study based in the Tampere University Hospital area, Finland, between 1 August 2021 and 1 April 2022.

### Setting

In the Tampere University Hospital area, emergency medical services (EMS) are produced by several service providers. They operate under the same administration inside the region that covers 15 600 km^2^ and has a population of 530 000 people. The annual number of EMS dispatches is 78 000.

The structure of Finnish EMS has previously been described in detail [11, 12]. The system is three-tiered, consisting of first responder units, nurse paramedic-staffed advanced level ambulances, and physician-staffed ambulance and helicopter emergency services. Common emergency dispatch is an ambulance. First responder units are used in a supporting role and sent to the scene automatically in some dispatches. A pre-hospital physician can be deployed in the field or consulted by ambulance.

Emergency services are routed through an emergency medical dispatcher (EMD). Dispatch urgency is classified by the EMD from A to D – A and B being immediately life threatening or more stable but urgent missions responded to with lights and sirens. Missions in the C-category are semi-urgent and D, non-urgent. The nurse paramedics are trained to assess the patient on scene and decide whether the patient needs immediate medical treatment or transportation. In addition, nurse paramedics reassess the urgency based on their evaluation. They can decide not to transport by ambulance if the patient’s condition does not warrant it, or the patient can be treated on scene. There is no standard protocol guiding nurse paramedics in the care of palliative patients in the study area.

The Finnish national guidelines and recommendations instruct physicians to recognize patients who need the goal of the treatment shifted to symptom-centered palliative care by making a palliative care decision. This decision is documented by the International Classification of Diseases (ICD)-10 code Z51.5 (Palliative care) in the patient records. Annually, over 1500 patients receive a palliative care decision (ICD-10 code Z51.5) in Tampere University Hospital.

The palliative care unit of Tampere University Hospital leads the regional palliative care pathway in collaboration with the communities in the region. The pathway includes home care teams and community hospital wards as well as the Pirkanmaa Hospice, where patients with a palliative care decision may be admitted without a visit in the emergency room (ER) in case of unexpected deterioration of symptoms or other palliative care needs.

### Population

The study population was gathered based on the palliative care decisions in the patient records. We included patients who were set a goal of therapy as palliative intent by a diagnosis code Z51.5 in the ICD-10 between 1 August 2021 and 31 December 2021 and who died before 1 April 2022. We included only patients living in municipalities where the EMS used an electronic patient record system. These municipalities were Akaa, Lempäälä, Nokia, Ruovesi, Tampere, Urjala, Valkeakoski, Vesilahti, Virrat, and Ylöjärvi. Altogether, 70% of the people in the EMS catchment area live in these municipalities. From these patients, the electronic patient care records were retrieved from the period of four weeks before the setting of the diagnosis code Z51.5 to the day when the patient passed away. If at least one paramedic visit to the patient was reported between the diagnosis and time of death, the patient was included. With these criteria it was ensured that the included patient truly was recognized to be in the end-of-life stage of their illness. The number of patient contacts to the palliative care unit was also retrieved.

### Data and statistical analyses

From the study population, basic patient data were collected from the patient records. These included age, gender, current diagnoses, and information about the conditions in which the palliative care decision was set. Precise information about the EMS visits and treatment given was also recorded from the patient records. Paramedics have been instructed to record information about the palliative care decision in the patient records. It was considered that the paramedic had recognized the situation and was aware of the patient’s advanced care plan if the palliative care decision was mentioned in the recording. The recorded data were analyzed using IBM SPSS statistics version 29. Descriptive statistics were used to describe the data.

## Results

**Fig. 1:**
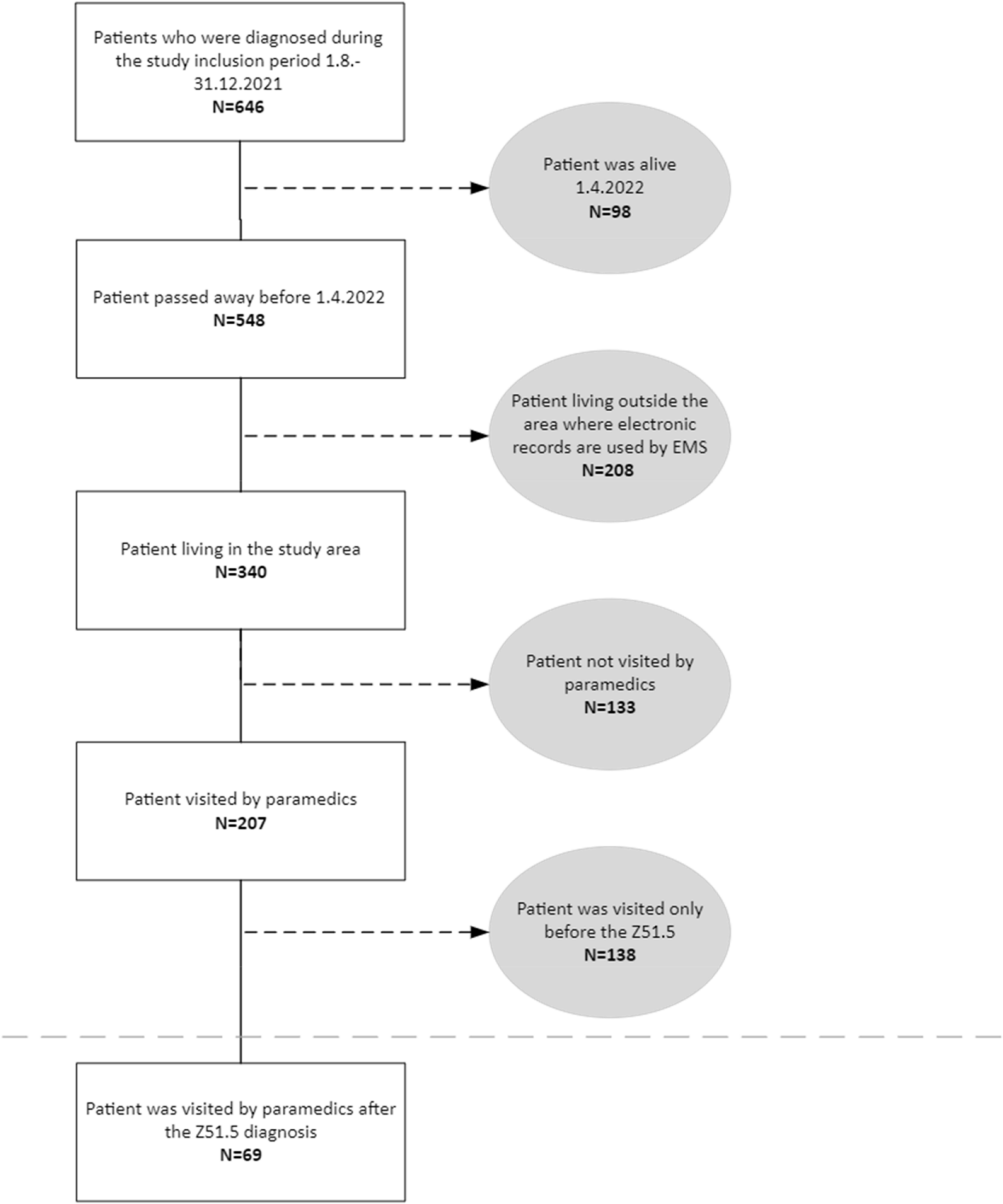
Patient selection process

There were 36 672 EMS dispatches in the study region during the study period. A palliative care diagnosis was established for 646 patients and 548 of them died before 1 April 1, 2022. Of these patients, 340 lived in the study area. Among them, the EMS visited 69 patients 97 times between the palliative diagnosis and death. Figure 1 describes the patient selection in detail. EMS dispatches towards palliative care patients formed 0.26% of the total dispatches in the study area during the study period. The most common location of the EMS dispatch was the patient’s home. Typically, the patient was visited once, but there were some patients who had several visits before death. Table 1 describes the patient characteristics. The most common dispatch codes were general weakness (25%), breathing difficulty (22%), pain (11%) and fall (9%). Urgent dispatches with lights and sirens (A and B) comprised 33% of all dispatches. Overall, 13% of the EMS dispatches led to transportation with lights and sirens. The most common dispatch and transportation codes are described in Table 2. After assessment by the EMS, 55% of the patients were transported to the emergency department. In 21% of the EMS missions, the patients were transported to a community hospital ward. The patient was transported in 76% of the dispatches. Paramedics provided treatment in 40% of the cases. The most common interventions were opening an intravenous line (30%), administering pain medication (21%), and providing supplemental oxygen (19%). Table 3 presents the need of patient transport, transport destination and treatment on scene. In one case the paramedics attempted resuscitation. A palliative care plan was recognized by paramedics in 42 of the 97 callouts. In 31 patients palliative care was not recognized during any visit, and in 38 patients it was recognized at least once. A total of 84% of the patients had established contact with the palliative care unit of Tampere University Hospital.

**Table 1:** Patient characteristics

Variable	
Patients *n*	69
Age *years*, *median, (Q1-Q3)*	76 (70–84)
Gender	
Male, *%*	52
Survival after palliative care decision, *months, median (Q1-Q3)*	2.0 (1.1–3.2)
Number of paramedic callouts, *n*	97
Number of visits per patient	
1	50 (73%)
2	13 (19%)
3	4 (6%)
4	1 (1%)
5	1 (1%)
Case location, *n*	
Home	79
Supported care	5
Ward	4
Other	9

**Table 2:** Reasons for paramedic response and transportation

	**Dispatch**	**Transportation**
Reason for paramedic visit, *%*		
General weakness	25%	35%
Breathing difficulty	22%	12%
Pain*	11%	6%
Fall	9%	3%
Other	42%	20%
Urgency		
A	12%	2%
B	21%	11%
C	28%	22%
D	39%	41%

^*^Other than chest pain

**Table 3:** Transportation and treatment during visit

Variable	
Number of EMS missions with any intervention *n (% of all missions)*	39 (40%)
Intravenous line placement *n (% of all missions)*	21 (30%)
Pain medication* n (% of all missions)*	15 (21%)
Supplemental oxygen* n (% of all missions)*	13 (19%)
Electrocardiography* n (% of all missions)*	6 (9%)
Other medication* n (% of all missions)*	5 (7%)
Continuous positive airway pressure* n (% of all missions)*	3 (4%)
Other	7 (10%)
Patients transported, *n (%)*	74 (76%)
Transport destination, *n (%)*	
Emergency department	53 (55%)
Community hospital ward or hospice	21 (21%)
No transportation	23 (24%)
Physician was consulted, *n (%)*	20 (21%)

## Discussion

EMS missions to palliative patients comprised less than 0.26% of all missions in the area and thus do not seem to contribute a significant load to the EMS. Nurse paramedics did not seem to recognize that the patients were in palliative care as efficiently as they should. This leads to unnecessary patient transportation, typically to the emergency department, which is commonly not beneficial and can be futile for the patients in palliative care. EMS interventions were not limited to symptom control.

Paramedic practice concerning palliative patients has been researched in larger cohort studies with similar findings concerning the mission characteristics, treatment provided and patient transportation [4, 6]. This study provides context by representing lack of patient recognition as a possible cause of inappropriate decisions made when the patient is in the palliative stage. In normal circumstances, paramedics are trained to provide lifesaving interventions to patients with life-threatening conditions. Symptoms in end-of-life patients often resemble serious medical conditions. Without knowledge about palliative care, the paramedics cannot make the right decisions about medical interventions and patient transportation. The lack of proper training among paramedics has been identified as a challenge [13]. In addition, time pressure and decision-making with insufficient information about the patient often lead to a situation in which paramedics see transportation to the emergency department as the only option [7, 8, 14].

Salminen et al. collected data on all EMS dispatches in the Tampere University Hospital area in August 2021, overlapping with our study period. This offers the possibility to directly compare missions towards palliative care patients to all the EMS callouts in the same population. Compared to a study by Salminen et al., the proportion of urgent dispatches (A or B) was similar in overall EMS callouts and palliative patients (33% vs. 36%) [15]. The number of urgent dispatches can be considered high because difficult symptoms are common in the palliative stage and, typically, a response with lights and sirens does not provide any actual benefit to the patient. In many cases, the paramedics assessed that the transportation of the palliative patient also needed to be urgent with lights and sirens (13%). This percentage was higher in palliative patients compared to all transported patients in the study region (13% vs. 8%) [16]. Urgent transportations always represent a risk to the ambulance personnel, patient and other people in traffic. This risk is not justified while transporting palliative patients because all treatment is limited to palliative symptom control.

In palliative patients, breathing difficulty is more often the reason for dispatch; it is the second-most common in palliative patients but only fourth in the whole population [16]. In the palliative stage, dyspnea is a common symptom in chronic obstructive pulmonary disease, congestive heart failure and cancer [17]. The high prevalence of breathing difficulties among palliative patients has also been reported in a larger Australian cohort [4]. In palliative symptom control, the treatment of dyspnea is similar regardless of the cause. This includes airflow to the face, medication with opioids and a semi-upright position. In the palliative stage, all more invasive treatment options are excluded. Nurse paramedics have the ability and training to treat these patients on scene, but to do so they would need to recognize palliative care patients because breathing difficulty is often an indication for urgent transportation in other patients.

In the Finnish setting, palliative care patients should have their advanced care plan available in their home in case of unexpected deterioration of symptoms. In case of an emergency, it is up to the patient or close relative to present this information to the paramedics. Considering that half of the palliative care patients were missed by the paramedics, it seems that existing information does not come up during the visit. A protocol for preplanned participation of paramedics in palliative care has been developed in the Finnish Northern-Karelia healthcare district. With this in place, patients were transported only in 56% of the EMS missions, and only 16% of the transferred patients are transported to a secondary hospital [6]. This shows that availability of the patient’s advanced care plan and proper training of the nurse paramedics can provide improvement.

In 2019 the Finnish Ministry of Social Affairs and Health gave a recommendation concerning palliative care [18]. It states that everyone has an equal right to adequate palliative care. The main concerns in achieving this goal were the lack of training among healthcare professionals and the high use of EMS among the aged population before the time of death. These challenges are also seen in our study in the form of high transportation to the emergency department and low percentage of palliative patients recognized. The number of palliative patients is expected to increase in the developed countries, including Finland, due to the aging population. This creates a practical and ethical need for improving EMS capability to treat palliative patients. Possible targets for development based on previous research and findings of this study could be improving the availability of patient records and training paramedics to actively seek and apply this information.

## Strengths and limitations

This study provides information from real-life EMS dispatches to palliative patients in a setting that has no protocol for EMS involvement in palliative care. The study design also enables recognition of possible challenges in the treatment of palliative patients by EMS. This study has limitations caused by the patient selection process. The patients’ palliative care diagnoses were retrieved from the records of Tampere University Hospital. This rules out palliative patients who have been treated only in primary healthcare. The number of these patients is unknown. It can be speculated that there are few of these patients because difficult symptoms often lead to referral to specialized healthcare. The number of patients in the palliative stage but not diagnosed with Z51.5 is also unknown. These cases, however, are probably quickly progressing situations that lead to patients’ death without the involvement of the EMS. The number of patients in the cohort is large enough to say that palliative patients form only a small minority of all EMS patients. Other findings must be interpreted with caution. It is unlikely that increasing the cohort size would significantly change the results.

## Conclusions

Patients in palliative care do not place a significant load on the EMS, but improvement is required in the way patients are treated. The high number of patients transported to the emergency department is against palliative care principles. The low number of palliative patients recognized provides a possible explanation for high patient transportation.

## Data Availability

The datasets used are available from the corresponding author on a reasonable request.

## Abbreviations

EMS: Emergency medical services
EMD: Emergency medical dispatcher
